# Delirium following mechanical thrombectomy for ischemic stroke – individuals at risk, imaging biomarkers and prognosis

**DOI:** 10.3389/fnagi.2025.1486726

**Published:** 2025-02-14

**Authors:** Marianne Hahn, Lavinia Brockstedt, Sonja Gröschel, Katharina Geschke, Nils F. Grauhan, Marc A. Brockmann, Ahmed E. Othman, Klaus Gröschel, Timo Uphaus

**Affiliations:** ^1^Department of Neurology, University Medical Center of the Johannes Gutenberg University Mainz, Mainz, Germany; ^2^Department of Neuroradiology, University Medical Center of the Johannes Gutenberg University Mainz, Mainz, Germany; ^3^Department of Psychiatry and Psychotherapy, University Medical Center of the Johannes Gutenberg University Mainz, Mainz, Germany

**Keywords:** stroke, mechanical thrombectomy, endovascular stroke therapy, delirium, atrophy, complications, prognosis

## Abstract

**Aim:**

Post-stroke-delirium has been linked to worse outcome in patients with acute cerebrovascular disease; identification of individuals at risk may prevent delirium and thereby improve outcome. We investigate prognosis and factors associated with post-stroke-delirium in patients with large vessel occlusion (LVO) ischemic stroke treated by mechanical thrombectomy (MT).

**Methods:**

747 patients (53.4% female) prospectively enrolled in the Gutenberg-Stroke-Study from May 2018–November 2022 were analyzed with regard to diagnosis of delirium. Group comparison of patient-, stroke- and treatment characteristics as well as computed tomography(CT)-imaging based parameters of cerebral atrophy (global cortical atrophy [GCA], posterior atrophy [Koedam], medial temporal lobe atrophy [MTA] scores) and white matter lesions (Fazekas score) was conducted. Independent predictors of delirium and the association of delirium with functional outcome at 90-day follow-up was investigated by multiple logistic regression analyses.

**Results:**

We report 8.2% of patients (61/747) developing delirium following MT of LVO. Independent predictors were older age (aOR[95%CI] per year: 1.034[1.005–1.065], *p* = 0.023), male sex (aOR[95%CI]: 2.173[1.182–3.994], *p* = 0.012), general anesthesia during MT (aOR[95%CI]: 2.455[1.385–4.352], *p* = 0.002), infectious complications (aOR[95%CI]: 1.845[1.031–3.305], *p* = 0.039), “other determined” etiology of stroke (aOR[95%CI]: 2.424[1.100–5.345], *p* = 0.028), and a MTA score exceeding age-specific cut-offs (aOR[95%CI]: 2.126[1.065–4.244], *p* = 0.033). Delirium was independently associated with worse functional outcome (aOR[95%CI]: 2.902[1.005–8.383], *p* = 0.049) at 90-day follow-up.

**Conclusion:**

Delirium is independently associated with worse functional outcome after MT of LVO, stressing the importance of screening and preventive measures. Besides conventional risk factors, pathological MTA scores and use of general anesthesia during MT may be easy-to-apply criteria to identify individuals at risk of delirium and implement prevention strategies.

## Introduction

Mechanical thrombectomy (MT) is a highly effective treatment for major ischemic stroke due to large vessel occlusion (LVO) and is now the standard of care for acute stroke treatment. However, patients who have suffered a major stroke are a group at high risk for developing complications during hospital stay (Shaw et al., 2019). A common complication in acute stroke patients is delirium, a syndrome characterized by an altered level of consciousness, disorganised thoughts or disorientation, psychomotor disturbances and circadian dysrhythmia, typically with acute onset and fluctuations over time. Delirium in stroke patients has been reported to be associated with worse outcomes (Shi et al., 2012).

Since treatment options are limited, delirium prevention is the goal for hospitalized patients. While interdisciplinary multicomponent interventions have proven effective for delirium prevention in the hospital setting (Burton et al., 2021), identification of individuals at risk of developing delirium may help to design more efficient and targeted preventive measures and enable timely diagnosis of delirium. Etiology of delirium is multifactorial and reports on risk factors vary throughout the literature with sometimes conflicting conclusions. Patient characteristics, including older age and premorbid cognitive decline; stroke characteristics, e.g., stroke location and severity; and further factors, such as infections, have all been reported to be associated with development of delirium in stroke patients (Rhee et al., 2022; Oldenbeuving et al., 2011; Hahn et al., 2023). Although clinical screening instruments for delirium in hospitalized patients have been developed, questions have been raised about their validity in patients with neurological deficits (Vater et al., 2024). Therefore, identification of risk factors and development of predictive tools may help to prevent and diagnose delirium in patients hospitalized due to acute stroke. The few predictive instruments that have been specifically developed for stroke patients largely remain to be externally validated (Drozdowska et al., 2021). Moreover, existing studies originate from mixed stroke cohorts, which predominantly consist of non-major stroke patients and include also patients with hemorrhagic stroke (Oldenbeuving et al., 2014). However, patients with LVO treated by MT constitute a distinct subgroup of stroke patients; data on delirium in these patients is sparse. These patients are poorly represented in published analyses of delirium, as mixed stroke cohorts contain few cases of MT or analyses were conducted before the era of MT as standard therapy for LVO (Shaw et al., 2019).

We investigate the association of patient-, stroke- and treatment characteristics with the development of delirium in a cohort of patients with LVO who were treated by MT. Additionally, we explore the role of easily obtainable imaging biomarkers of cerebral atrophy and white matter lesions (WML) arising from native computed tomography (CT) as a potential independent risk factor for developing delirium. Finally, we analyze whether delirium is an independent predictor for functional outcome three months after MT of LVO.

## Methods

### Study cohort and outcome parameters

The Gutenberg-Stroke-Study is an ongoing monocentric, prospective, observational study that consecutively enrolls adult patients diagnosed with acute ischemic stroke in our certified German University Hospital stroke center. All 747 patients with LVO treated by MT enrolled in the Gutenberg-Stroke-Study from May 2018 to November 2022 were included in our primary analysis. Baseline demographics, cardiovascular comorbidities, clinical and procedural information as well as clinical follow-up after 90 days (carried out by standardized telephone interview) are recorded. At 90-day follow-up, good functional outcome was defined as modified Rankin Scale score (mRS) ≤2 or mRS equal to premorbid mRS in case of premorbid mRS >2. Excellent functional outcome was defined as mRS ≤1 or mRS equal to premorbid mRS in case of premorbid mRS >1. Cognitive outcome was evaluated by telephone-based Montreal Cognitive Assessment (MoCA) (Nasreddine et al., 2005). Patients in our comprehensive stroke center are screened for delirium three times a day by stroke-unit nurses on the basis of the Nursing Delirium Screening Scale (NU-DESC) (Lütz et al., 2008) as specified in the local standard operating procedure. Diagnosis of delirum was then obained by the treating stroke-unit physician. We extracted diagnosis of delirium during the hospital stay based on diagnosis as classified by the International Statistical Classification of Disease and related health problems – 10th revision (ICD-10) used for financial reimbursement from the healthcare providers. Possible confounders of delirium (premorbid dementia; acute infections such as pneumonia, urinary tract infection and/or sepsis) were also extracted by diagnosis as classified by the ICD-10.

### Standard protocol approval and data availability

Study protocols and procedures were conducted in compliance with the Declaration of Helsinki and in accordance with local ethical guidelines. The Gutenberg-Stroke-Study was approved by the responsible ethics committee of the Landesärztekammer Rheinland-Pfalz (approval number: 2018-13335-Epidemiologie). Written informed consent was obtained from all participants (or guardians of participants). The Gutenberg-Stroke-Study is registered in the German Clinical Trial Registry (DRKS00017253). The manuscript follows the STROBE guideline. The data supporting the findings of this study are available from the corresponding author on reasonable request from any qualified investigator.

### Imaging biomarkers of cerebral atrophy and white matter lesions

Native CT scans from primary admission for stroke evaluation prior to acute stroke treatment were rated by trained neuroradiology physicians blinded from outcome parameters. Assessment included parameters of cerebral atrophy, namely global cortical atrophy score (GCA, ranging from 0 to 3 with 3 representing severe atrophy) (Pasquier et al., 1996), posterior atrophy score (PA, Koedam, ranging from 0 to 3 with 3 representing severe atrophy) (Koedam et al., 2011), and medial temporal lobe atrophy score (MTA, ranging from 0 to 4 with 4 representing end stage atrophy) (Scheltens et al., 1992). Burden of WML was assessed by Fazekas score (ranging from 0 to 3 with 3 representing the highest burden of WML) (Fazekas et al., 1987). Taking into account normative values of brain atrophy (Cotta Ramusino et al., 2019; Rhodius-Meester et al., 2017), age-specific cut-offs for pathological score values were set as follows: pathological GCA <75 years: ≥1, ≥75 years: ≥2; pathological PA <65 years: ≥1, ≥65 years: ≥2; pathological MTA <65 years: ≥1, 65–79 years: >2, ≥80 years: ≥3; pathological Fazekas <65 years: ≥1, 65–74 years: ≥2, ≥75 years: 3.

### Statistical analysis

The study cohort was divided into two comparison groups depending on diagnosis of delirium during hospital stay following MT. Statistical analyses were carried out on the basis of complete datasets for the respective outcome parameter. Data is presented as mean ± SD, median (interquartile range, IQR) or proportions (categorical variables), unless indicated otherwise. Number of available observations is stated for each variable. To identify patient-, stroke- and treatment characteristics associated with delirium, group comparison on univariate level was performed by Mann–Whitney-U test, chi-square test or Fishers exact test as appropriate. To investigate independent predictors of delirium and a potential predictive capacity of imaging parameters of neurodegeneration, a multiple logistic regression analysis was conducted. Variables were entered blockwise with demographic patient characteristics (age, sex) and variables significantly differing on univariate level in the first block, followed by a second block with stepwise variable selection by backward elimination containing scores on atrophy (GCA, Koedam, MTA), WML (Fazekas) and premorbid clinical diagnosis of dementia, to investigate a potential additional predictive power of these variables. Analysis of the area under the receiver operating characteristic (ROC) curve (AUC) was performed to assess predictive power of the regression model.

To assess predictive capacity of an existing screening instrument developed by Oldenbeuving et al. (2014) to identify acute stroke patients with high risk of delirium, we accordingly calculated their risk score ‘Model 2’ (containing age, National Institute of Health Stroke Scale [NIHSS] on admission, stroke subtype and presence of infection) and ‘Model 3’ (containing only age and NIHSS on admission) for patients in our cohort and conducted an AUC comparison between our multiple regression model and the proposed scoring system.

To assess an independent association of delirium with functional/cognitive outcome, multiple logistic regression analysis (functional outcome) and multiple linear regression analysis (cognitive outcome) was performed. The models adjust for the following confounders, selected by significant differences on univariate level and literature-based predictors of outcome following MT: age, sex, premorbid disability (mRS > 2), NIHSS on admission, intravenous thrombolysis, general anesthesia during MT, successful recanalization (thrombolysis in cerebral infarction scale score [TICI] 2b-3), time from admission to flow restoration, stroke etiology and pneumonia. Linearity was assessed using the Box-Tidwell procedure. All continuous variables were found to follow a linear relationship. For multicollinearity diagnostics, we calculated variance inflation factors, assuming no relevant multicollinearity for values <10. Predictive capacity of the multiple logistic regression models was assessed by Nagelkerke’s R^2^. Goodness of fit was assessed using the Hosmer-Lemeshow-Test, indicating a good model fit for *p* > 0.05. Multiple regression modelling was performed on the basis of complete datasets on the analyzed outcome and predictor variables. A significant difference was considered for *p* < 0.05 in all analyses. Statistical analyses were performed using SPSS (version 29, IBM, Armonk, NY, United States) and MedCalc Statistical Software (version 19.6, MedCalc Software Ltd., Ostend, Belgium).

## Results

### Univariate predictors of delirium following mechanical thrombectomy and associated outcome parameters

#### Baseline-, stroke- and treatment characteristics

**Figure 1:**
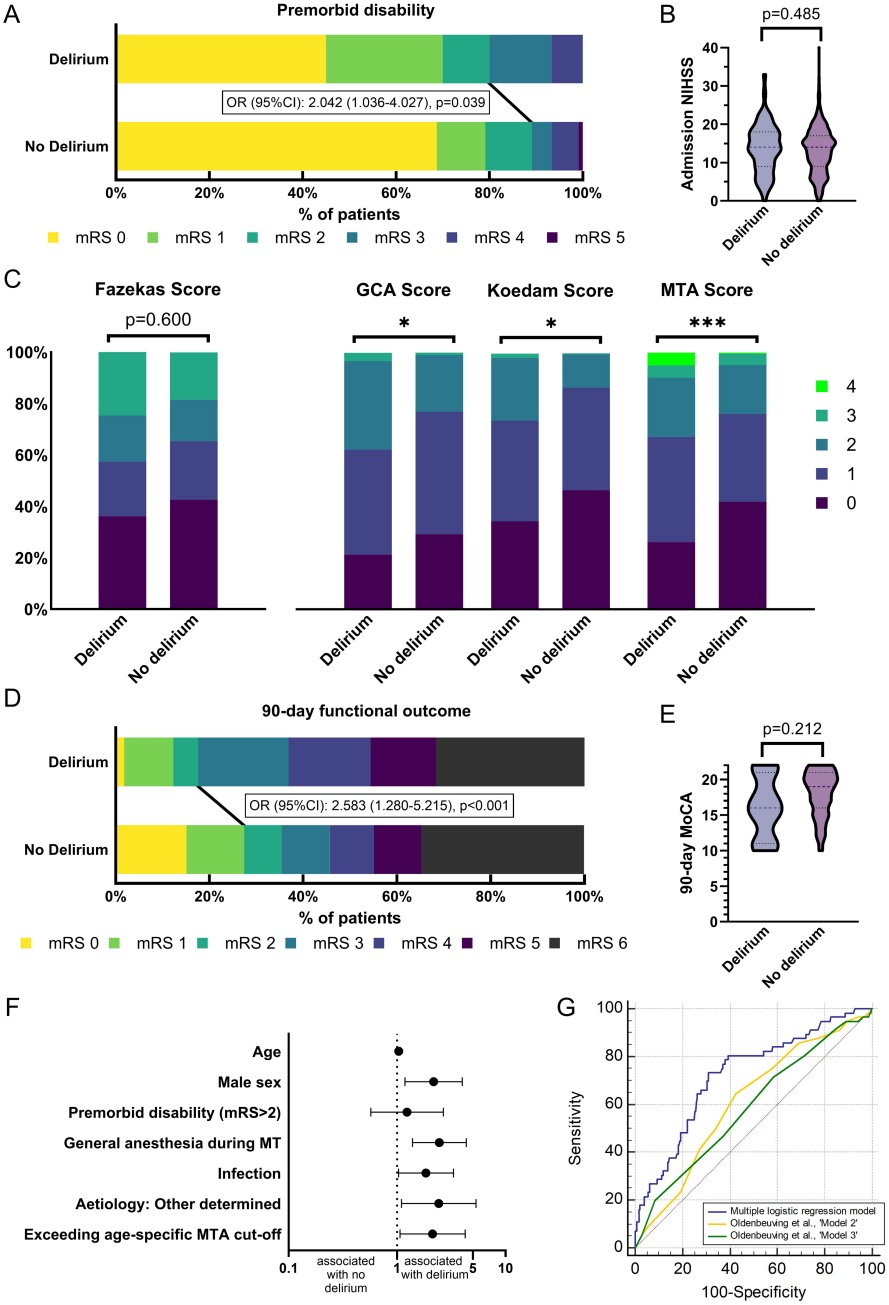
Outcomes and determinants of delirium in patients treated by mechanical thrombectomy. **(A)** Distribution of premorbid mRS in patients with and without delirium. Patients with delirium have significantly higher premorbid disability. **(B)** No significant differences in stroke severity measured by NIHSS on admission in patients with and without delirium. **(C)** White matter lesion burden (Fazekas) does not differ between patients with and without delirium. Distribution of all cerebral atrophy scores (GCA, Koedam, MTA) is significantly different in patients with versus without delirium. **(D)** Distribution of mRS at 90-day follow-up in patients with and without delirium. Patients with delirium have significantly worse functional outcome. **(E)** No significant differences in cognitive outcome (MoCA) at 90-day follow-up in patients with and without delirium. **(F)** Independent predictors of delirium following MT of LVO. Displayed are odds ratios with 95%CI resulting from multiple logistic regression analysis. **(G)** AUC analysis of multiple logistic regression model yields significant predictive capacity of delirium following MT of LVO. In AUC comparison, predictive capacity is significantly higher than with previously developed tools for risk stratification for delirium in stroke patients by Oldenbeuving et al. (2014). Asterisks: *p*-values indicating significant difference under a threshold of *: <0.05; ***: <0.001. AUC: Area under the receiver operating characteristic curve, GCA: global cortical atrophy score, Koedam: posterior atrophy score, LVO: large vessel occlusion, MoCA: Montreal Cognitive Assessment, MT: mechanical thrombectomy, MTA: medial temporal lobe atrophy score, NIHSS: National Institute of Health Stroke Scale, IVT: intravenous thrombolysis, mRS: modified Rankin Scale score.

Our analysis included 747 patients (median age 77.0 years, 53.4% female, Table 1). Of these, 8.2% (*n* = 61) were diagnosed with delirium during their hospital stay following MT of LVO. In univariate group comparison, patients with delirium were more often male (60.7% versus 45.3%, *p* = 0.022) and had more often relevant premorbid disability, measured by pre-stroke mRS > 2 (20.0% [12/60] versus 10.9% [71/673]; *p* = 0.035, Figure 1A). There was no significant difference in cardiovascular risk factor burden, nor did we note a significant difference in premorbid clinical diagnosis of dementia in patients with or without delirium (6.6% versus 4.1%, *p* = 0.360). Stroke severity, measured by NIHSS on admission (see also Figure 1B), and vessel territory of LVO were also similar. With regard to stroke etiology, we report more strokes classified as “other determined” etiology in patients with delirium (20.0% [12/60] versus 9.0% [59/656], *p* = 0.006). Treatment characteristics were similar between groups with regard to application of bridging intravenous thrombolysis, rates of referral for MT from another hospital, treatment times and rates of successful reperfusion, defined by TICI 2b-3. Rates of general anesthesia use during MT were much higher in patients developing delirium (50.9% [29/57] versus 30.8% [200/649], *p* = 0.002). Patients diagnosed with delirium more often had acute infectious complications (62.3% versus 42.7%, *p* = 0.003), encompassing pneumonia, urinary tract infection and/or sepsis. Especially pneumonia was more common in patients with delirium (50.8% versus 34.0%, *p* = 0.008).

**Table 1:** Patient-, stroke- and treatment characteristics in patients with and without developing delirium following mechanical thrombectomy of large vessel occlusion ischemic stroke.

Variable	No delirium (*n* = 686)	Delirium (*n* = 61)	*p* value
Patient characteristics			
Age (years)	77 (65–83) (*n* = 686)	79 (69.5–84.5) (*n* = 61)	0.176
Male Sex	45.3% (311/686)	60.7% (37/61)	**0.022**
Premorbid disability (mRS 3–5)	10.9% (71/652)	20.0% (12/60)	**0.035**
History of dementia	4.1% (28/686)	6.6% (4/61)	0.360
Cardiovascular risk factors			
Arterial hypertension	78.3% (527/673)	80.0% (48/60)	0.760
Diabetes mellitus	27.5% (183/665)	32.2% (19/59)	0.442
Dyslipidemia	44.2% (293/663)	38.3% (23/60)	0.381
Atrial fibrillation	43.2% (288/667)	46.6% (27/58)	0.619
Smoker (current)	14.3% (80/559)	24.0% (12/50)	0.067
Stroke characteristics			
NIHSS on admission	14 (9–17) (*n* = 637)	14 (9–18) (*n* = 57)	0.485
Presenting with aphasia	45.6% (313/686)	42.6% (26/61)	0.652
Location of occlusion			
Carotid artery	21.2% (145/683)	21.3% (13/61)	0.988
Anterior cerebral artery	2.9% (20/683)	1.6% (1/61)	1.000
Middle cerebral artery M1 segment	52.6% (359/683)	44.3% (27/61)	0.214
Middle cerebral artery M2 segment	26.9% (184/683)	32.8% (20/61)	0.327
Posterior cerebral artery	3.1% (21/683)	3.3% (2/61)	0.712
Vertebrobasilar	11.4% (78/683)	14.8% (9/61)	0.438
Side of occlusion			0.681
Left	43.5% (297/682)	36.1% (22/61)	
Right	46.2% (315/682)	52.5% (32/61)	
Bilateral	0.3% (2/682)	0.0% (0/61)	
Not applicable (e.g., BA)	10.0% (68/682)	11.5% (7/61)	
Stroke etiology			
Large artery atherosclerosis	16.0% (105/656)	8.3% (12/60)	0.115
Cardioembolism	50.5% (331/656)	51.7% (31/60)	0.858
Dissection	1.8% (12/656)	3.3% (2/60)	0.331
Other determined	9.0% (59/656)	20.0% (12/60)	**0.006**
Undetermined	22.7% (149/656)	16.7% (10/60)	0.281
Treatment characteristics			
Intravenous thrombolysis	47.9% (325/679)	55.0% (33/60)	0.289
Primary admission at MT site	59.4% (401/675)	57.6% (34/59)	0.790
Symptom onset/Time of recognition-to-admission (minutes)	108.5 (60–190) (*n* = 594)	90 (60–205) (n = 53)	0.960
Door-to-groin puncture (minutes)	60 (31–87) (*n* = 616)	57.5 (28–95.25) (*n* = 54)	0.992
General anesthesia used	30.8% (200/649)	50.9% (29/57)	**0.002**
Successful reperfusion (TICI 2b-3)	81.4% (540/663)	88.1% (52/59)	0.200
Infectious complications			
Acute infection (total of below)	42.7% (293/686)	62.3% (38/61)	**0.003**
Pneumonia	34.0% (233/686)	50.8% (31/61)	**0.008**
Urinary tract infection	13.7% (94/686)	18.0% (11/61)	0.351
Sepsis	3.1% (21/686)	3.3% (2/61)	0.711

Data are presented as percentage (absolute number) except for age, NIHSS on admission, symptom onset/time of recognition-to-admission, door-to-groin puncture: median (IQR) (number of available observations). mRS: modified Rankin Scale; MT: mechanical thrombectomy; NIHSS: National Institutes of Health Stroke Scale; TICI: Thrombolysis in cerebral infarction scale.

#### Atrophy patterns and white matter lesions on native CT

The distribution of all three scoring systems on cerebral atrophy (GCA, Koedam, MTA) significantly differed between study groups (Figure 1C; Table 2). The proportion of patients exceeding age-specific cut-off values for cerebral atrophy was significantly higher in patients with delirium (GCA: 54.1% versus 38.5%. *p* = 0.015; Koedam: 31.1% versus 16.4%, *p* = 0.004; MTA: 24.6% versus 11.7%, *p* = 0.004). WML burden was similar with regard to distribution between groups as well as proportion of patients exceeding age-specific cut-offs.

**Table 2:** Atrophy patterns and white matter lesions in patients with and without delirium.

Variable	No delirium (*n* = 686)	Delirium (*n* = 61)	*p* value
Global cortical atrophy score (GCA)			
Absent	29.3% (200/683)	21.3% (13/61)	**0.037**
Grade 1	47.7% (326/683)	41.0% (25/61)	
Grade 2	22.1% (151/683)	34.4% (21/61)	
Grade 3	0.9% (6/683)	3.3% (2/61)	
Exceeding age-specific cut-off	38.5% (263/683)	54.1% (33/61)	**0.015**
Posterior atrophy score (Koedam)			
Absent	46.4% (317/683)	34.4% (21/338)	**0.032**
Grade 1	40.1% (274/683)	39.3% (24/61)	
Grade 2	13.0% (89/683)	24.6% (15/61)	
Grade 3	0.4% (3/683)	1.6% (1/61)	
Exceeding age-specific cut-off	16.4% (112/683)	31.1% (19/61)	**0.004**
Medial temporal lobe atrophy score (MTA)			
Absent	41.9% (286/683)	26.5% (16/61)	**<0.001**
Grade 1	34.3% (234/683)	41.0% (25/61)	
Grade 2	19.0% (130/683)	23.0% (14/61)	
Grade 3	4.4% (30/683)	4.9% (3/61)	
Grade 4	0.4% (3/683)	4.9% (3/61)	
Exceeding age-specific cut-off	11.7% (80/683)	24.6% (15/61)	**0.004**
White matter lesions (Fazekas score)			
Absent	42.6% (291/683)	36.1% (22/61)	0.600
Grade 1	22.8% (156/683)	21.3% (13/61)	
Grade 2	16.1% (110/683)	18.0% (11/61)	
Grade 3	18.4% (126/683)	24.6% (15/61)	
Exceeding age-specific cut-off	23.9% (163/683)	27.9% (17/61)	0.484

Data are presented as percentage (absolute number).

#### Functional and cognitive outcome parameters

Patients with delirium had a longer duration of hospital stay and worse functional outcome at 90-day follow-up (Table 3). Good functional outcome was less frequent in patients with delirium (19.3% [11/57] versus 37.1% [226/609], *p* = 0.007, Figure 1D) as was excellent outcome. Mortality until 90-day follow-up was similar in both groups (31.6% [18/57] versus 34.5% [210/609], *p* = 0.659). With regard to cognitive outcome, measured by MoCA at 90-day follow-up, we report no significant difference between groups (median [IQR]: 16 [11–21] versus 19 [16–20], *p* = 0.212, Figure 1E).

**Table 3:** Outcome parameters in patients with and without developing delirium following mechanical thrombectomy of large vessel occlusion ischemic stroke.

Outcome parameter	No delirium (*n* = 686)	Delirium (*n* = 61)	*p* value
Duration of hospital stay (days)	9 (5–13) (*n* = 679)	13 (7.25–17) (*n* = 60)	**<0.001**
Excellent outcome at 90-day follow-up (mRS ≤1 or as pre-mRS)	29.4% (179/609)	14.0% (8/57)	**0.014**
Good outcome at 90-day follow-up (mRS ≤2 or as pre-mRS)	37.1% (226/609)	19.3% (11/57)	**0.007**
90-day mortality	34.5% (210/609)	31.6% (18/57)	0.659
MoCA at 90-day follow-up	19 (16–20) (*n* = 210)	16 (11–21) (*n* = 11)	0.212

Data are presented as percentage (absolute number) except for duration of hospital stay, MoCA: median (IQR) (number of available observations). mRS: modified Rankin Scale; MoCA: Montreal Cognitive Assessment.

### Independent predictors of delirium following mechanical thrombectomy

Resulting from multiple logistic regression modelling, independent predictors of delirium were older age (aOR[95%CI] per year: 1.034[1.005–1.065], *p* = 0.023), male sex (aOR[95%CI]: 2.173[1.182–3.994], *p* = 0.012), general anesthesia during MT (aOR[95%CI]: 2.455[1.385–4.352], *p* = 0.002), infectious complications (aOR[95%CI]: 1.845[1.031–3.305], *p* = 0.039), and “other determined” etiology of stroke (aOR[95%CI]: 2.424[1.100–5.345], *p* = 0.028). Regarding atrophy scores (GCA, Koedam, MTA), Fazekas score and pre-existing clinical diagnosis of dementia, only MTA exceeding age-specific cut-offs emerged from the model as an independent predictor of delirium (aOR[95%CI]: 2.126[1.065–4.244], *p* = 0.033, see also Figure 1F). For detailed model characteristics, see also Table 4.

**Table 4:** Independent predictors of delirium and independent association of delirium with functional and cognitive outcome at 90-day follow-up.

Independent predictors of delirium resulting from multiple logistic regression modelling Model characteristics: Nagelkerkes R^2^ = 0.127, *p* < 0.001			
Variable	Adjusted odds ratio	95% CI	*p*-value
Age	1.034	1.005–1.065	**0.023**
Male sex	2.173	1.182–3.994	**0.013**
Premorbid disability (mRS 3–5)	1.236	0.572–2.674	0.590
General anesthesia during MT	2.455	1.385–4.352	**0.002**
Infectious complication	1.845	1.031–3.305	**0.039**
Etiology: “other determined”	2.424	1.100–5.345	**0.028**
Exceeding age-specific MTA cut-off	2.126	1.065–4.244	**0.033**

Independent association of delirium with functional and cognitive outcome at 90-day follow-up			
Variable	Adjusted odds ratio/β-coefficient	95% CI	*p*-value
Not achieving excellent outcome at 90-day follow-up (mRS ≤ 1 or as premorbid mRS)	3.440	1.045–11.327	**0.042**
Not achieving good outcome at 90-day follow-up (mRS ≤ 2 or as premorbid mRS)	2.902	1.005–8.383	**0.049**
90-day mortality	0.773	0.321–1.859	0.565
MoCA at 90-day follow-up	−0.242	−2.557-2.074	0.837

Point estimates, 95% CI and corresponding *p*-values resulting from multiple logistic/linear regression modelling. The multiple regression models assessing independent association of delirium with functional/cognitive outcome adjust for the following confounders: age, sex, premorbid disability (mRS > 2), NIHSS on admission, intravenous thrombolysis, general anesthesia during MT, successful recanalization (thrombolysis in cerebral infarction scale score [TICI] 2b-3), time from admission to flow restoration, stroke etiology and pneumonia. mRS: modified Rankin Scale; MT: mechanical thrombectomy; MTA: Medial temporal lobe atrophy score; MoCA: Montreal Cognitive Assessment.

### Predictive capacity of multiple regression model and comparison with published scoring system for delirium prediction in stroke patients

In ROC analysis, the multiple regression model yielded significant predictive capacity with an AUC [95%CI] of 0.725[0.690–0.759] (*p* < 0.001, Figure 1G). Applying the proposed scoring instrument of Oldenbeuving et al. (2014) to our dataset, we report an AUC [95%CI] of 0.611[0.573–0.648] for their ‘model2’ (containing age, NIHSS on admission, stroke subtype and presence of infection) in our cohort of patients with LVO treated by MT. Their simplified score ‘model3’ (containing only age and NIHSS on admission) resulted in an AUC [95%CI] of 0.588[0.549–0.625]. Thus, both models performed significantly worse than our multiple regression model in prediction of delirium following MT of LVO (*p* = 0.005 for ‘model2’, *p* = 0.006 for ‘model 3’).

### Independent association of delirium with worse functional outcome

Adjusting for differences between study groups and literature-based predictors of outcome after MT of LVO, we report an independent association of delirium with worse functional outcome. Patients with delirium were less likely to achieve good functional outcome (aOR[95%CI]: 2.902[1.005–8.383], *p* = 0.049) or excellent functional outcome (aOR[95%CI]: 3.440[1.045–11.327], *p* = 0.042). We did not observe an independent association of delirium with mortality or cognitive outcome. For details, see Table 4.

## Discussion

We here show that delirium is a complication associated with worse functional outcome in patients with LVO who were treated by MT and present data on conventional and novel predictive factors for development of delirium in these patients, which help to identify patients at risk and implement specific preventive measures to improve clinical outcome.

With regard to functional outcome, our results are in line with previous analyses of delirium in patients with acute stroke. These reported worse functional outcome in mixed stroke cohorts, mostly focusing on mortality and functional dependence at hospital dicharge (Shi et al., 2012; Rollo et al., 2022; Pasińska et al., 2019). Patients with LVO treated by MT were largely underrepresented in former studies of delirium in acute stroke and yet, depict a relevant share of the disease. We expand former findings by showing that also in stroke patients with LVO treated by MT delirium is associated with worse functional outcome at discharge. This finding persists three months after stroke and, notably, is independent of stroke severity. We consider this remarkable, especially since functional outcome in patients with major ischemic stroke due to LVO is mainly driven by severity of the disease. In contrast to former studies of delirium in stroke patients, we did not observe increased mortality in patients with LVO who developed delirium, which is likely due to high overall mortality rates attributable to other complications and severity of the disease. Increased hospital stay duration has been reported in patients with delirium in mixed stroke cohorts (Shi et al., 2012). This was also true for our patients treated by MT. This is notable, because in this cohort, hospital discharge is often determined by stroke-associated neurological deficits affecting independence in daily activities and self-care.

Our analyses identify patient-, stroke- and treatment characteristics that are associated with development of delirium in patients treated by MT. These have direct clinical implications by enabling identification of patients at risk for delirium to target preventive measures or contribute to early diagnosis. As expected, older age and infectious complications were independently associated with delirium following MT. These are known risk factors for delirium in hospitalized patients and, as shown previously, in patients with acute stroke (Oldenbeuving et al., 2011). We observed male sex to be independently associated with development of delirium following MT. This is especially interesting and merits further investigation, since prospective validated prediction models do not describe sex as a risk factor for delirium (Inouye et al., 2014; Zaal et al., 2015). Potentially, a difference in motor subtypes of delirium could contribute to our observation. Supporting this, a recent meta-analysis reports that hypoactive cases of delirium were more likely to be female (Ghezzi et al., 2022). Consequently, this might lead to increased diagnosis of delirium in men in observational routine care data, especially since persisting neurological deficits further impede diagnosis of delirium following MT of LVO (Vater et al., 2024).

Premorbid cognitive decline and cerebral atrophy have been repeatedly reported to be associated with development of delirium in patients with acute stroke. Investigation of the predictive capacity of cerebral atrophy for delirium in these patients, independent of conventional risk factors, has yielded conflicting results (Oldenbeuving et al., 2011; Rhee et al., 2022; Czyzycki et al., 2021; Qu et al., 2018). We show that a pathologic MTA score may be an easily accessible biomarker in native CT imaging that is independently associated with a 2-fold increase of odds for development of delirium following MT. Pathological MTA scores are associated with cognitive decline and Alzheimer’s disease (Scheltens et al., 1992). However and interestingly, premorbid clinical diagnosis of dementia was neither more frequent in patients with delirium nor adding predictive value to multiple regression modelling of delirium in our cohort. Increased awareness among stroke-unit personnel for the need of delirium prevention in patients with known dementia and consecutively better preventive measures may have potentially contributed to absence of increased delirium rates in these patients. However, it is more likely that this observation may be due to underdiagnosis of dementia or early phases of cognitive decline, not yet diagnosed as dementia, but apparent as brain atrophy. Similarly, underreporting of dementia diagnosis when obtaining medical history from a third party, as is often the case for patients with major ischemic stroke treated by MT, may contribute to our observation. Consequentially, pathological MTA scores, as an objective CT imaging biomarker associated with cognitive decline may provide additional predictive benefit instead.

With regard to treatment characteristics, the use of general anesthesia during MT was the only procedural feature independently associated with development of delirium. We report an almost 2.5-fold increase of odds to develop delirium following MT under general anesthesia. This finding is new in patients with LVO treated by MT and is clearly supported by systemic reviews reporting strong evidence for mechanical ventilation as a precipitating factor for delirium in the intensive care unit (Zaal et al., 2015). The role of anesthesia type during MT in functional outcome is controversially discussed (Sarraj et al., 2023; Chabanne et al., 2023; Campbell et al., 2023). Although further investigation is warranted, our finding demonstrates that delirium is a potential complication associated with use of general anesthesia during MT and argues against its across-the-board use in the management of LVO MT. Future studies should examine, whether patients developing delirium after MT benefit less from general anesthesia during MT, and, whether risk stratification for benefit of general anesthesia during MT could be improved by including independent risk factors for developing delirium.

Only few predictive tools exist to identify individuals with acute stroke at high risk of developing delirium (Drozdowska et al., 2021). Even though internal validation of these tools yielded comparatively high predictive capacity within the small mixed stroke cohorts they were derived from, it is questionable whether such tools are applicable to patients with LVO treated by MT. Such developed tools only provided limited predictive power in our cohort of MT-treated patients. We suggest that this is partly due to high weights placed on stroke severity in published predictive tools. This appears to enable identification of individuals at risk in mixed stroke cohorts, yet might be of limited significance in cohorts of patients with major stroke due to LVO. In line with this, we observed no significant difference in NIHSS on admission in patients with and without delirium in our cohort. Additional factors associated with delirium in MT-treated patients may enhance risk stratification in this distinct subgroup of acute stroke patients and should be considered in risk assessment for developing delirium in these patients.

Despite analyzing a broad dataset of patient-, stroke- and treatment characteristics, our study approach has several limitations. We observed a comparatively low incidence of delirium (8.2%) in our study cohort. The literature is inconsistent with a broad range regarding delirium incidence following acute stroke, yet observational data from routine care, as is our dataset, might be prone to underdiagnosis of delirium. Especially hypoactive phenotypes of the disease might be underdiagnosed. The implementation of screening for delirium on a regular basis, as has recently been added to national guidelines of acute stroke care (Ringleb et al., 2022), might help to overcome this potential source of bias in the future and allow for even more accurate analyses of delirium from observational data. Furthermore, reduced sensitivity for diagnosis of delirium has been shown for ICD-10 diagnoses, e.g., compared to discharge reports (Chuen et al., 2022; Thomas et al., 2012), which might further explain the comparatively low delirium incidence in our dataset. Due to the nature of our monocentric study cohort, our findings are also limited with regard to transferability and generalizability. Our analysis might also have limited generalizability to populations with an ethnical distribution differing from the western European one. Future studies should confirm and further examine our findings.

Our analysis is based on a well-characterized large cohort of patients with major stroke due to LVO treated by MT. Therefore, we were able to investigate a comprehensive set of stroke- and treatment characteristics, potentially associated with delirium. As delirium has previously only been analyzed in mixed stroke cohorts with few cases treated by MT, our findings are new and highly relevant for acute care of these patients. Furthermore, we identify risk factors and an imaging biomarker in native CT that are easy to obtain in clinical practice. This is an important advantage with regard to transferability into clinical practice for identification of individuals at high risk of developing delirium following MT.

## Conclusion

We demonstrate that previously developed tools for identifying stroke patients at high risk for delirium have only limited predictive capacity in a cohort of patients treated by MT. Besides conventional risk factors such as age and infectious complications, additional risk factors for delirium exist that were not included in tools for risk stratification in mixed stroke cohorts until now. A pathologic MTA score, rated in native CT, may be an easy to use imaging biomarker identifying patients at increased risk for delirium following MT as is the use of general anesthesia during the MT procedure. Prevention of delirium is our best therapeutic strategy, as therapy options, once incident, are limited. Identification of individuals at high risk may help to intensify preventive measures and allow for a targeted resource allocation. The fact that delirium is associated with worse functional outcome, even in our cohort of severely diseased patients with LVO, justify and stress the importance of increased efforts to predict and prevent this complication.

## Data Availability

The raw data supporting the conclusions of this article will be made available by the authors, without undue reservation.

## References

[ref1] Burton J. K. Craig L. E. Yong S. Q. Siddiqi N. Teale E. A. Woodhouse R.. (2021). Non-pharmacological interventions for preventing delirium in hospitalised non-ICU patients. Cochrane Database Syst. Rev. 7:CD013307. doi: 10.1002/14651858.CD013307.pub2, PMID: 34280303 PMC8407051

[ref2] Campbell D. Butler E. Campbell R. B. Ho J. Barber P. A. (2023). General anesthesia compared with non-GA in endovascular Thrombectomy for ischemic stroke: a systematic review and Meta-analysis of randomized controlled trials. Neurology 100, e1655–e 1663. doi: 10.1212/WNL.0000000000207066 36797071 PMC10115505

[ref3] Chabanne R. Geeraerts T. Begard M. Balança B. Rapido F. Degos V.. (2023). Outcomes after endovascular therapy with procedural sedation vs general anesthesia in patients with acute ischemic stroke: the AMETIS randomized clinical trial. JAMA Neurol. 80, 474–483. doi: 10.1001/jamaneurol.2023.0413, PMID: 37010829 PMC10071397

[ref4] Chuen V. L. Chan A. C. H. Ma J. Alibhai S. M. H. Chau V. (2022). Assessing the accuracy of international classification of diseases (ICD) coding for delirium. J. Appl. Gerontol. 41, 1485–1490. doi: 10.1177/07334648211067526, PMID: 35176883 PMC9024024

[ref5] Cotta Ramusino M. Altomare D. Bacchin R. Ingala S. Bnà C. Bonetti M.. (2019). Medial temporal lobe atrophy and posterior atrophy scales normative values. Neuroimage Clin. 24:101936. doi: 10.1016/j.nicl.2019.101936, PMID: 31382240 PMC6690662

[ref6] Czyzycki M. Glen A. Slowik A. Chrzan R. Dziedzic T. (2021). Clinical utility of brain computed tomography in prediction of post-stroke delirium. J. Neural. Transm. (Vienna) 128, 207–213. doi: 10.1007/s00702-020-02294-9, PMID: 33417010

[ref7] Drozdowska B. A. McGill K. McKay M. Bartlam R. Langhorne P. Quinn T. J. (2021). Prognostic rules for predicting cognitive syndromes following stroke: a systematic review. Eur. Stroke J. 6, 18–27. doi: 10.1177/2396987321997045, PMID: 33817331 PMC7995322

[ref8] Fazekas F. Chawluk J. B. Alavi A. Hurtig H. I. Zimmerman R. A. (1987). MR signal abnormalities at 1.5 T in Alzheimer's dementia and normal aging. AJR Am. J. Roentgenol. 149, 351–356. doi: 10.2214/ajr.149.2.351, PMID: 3496763

[ref9] Ghezzi E. S. Greaves D. Boord M. S. Davis D. Knayfati S. Astley J. M.. (2022). How do predisposing factors differ between delirium motor subtypes? A systematic review and meta-analysis. Age Ageing 51, 1–13. doi: 10.1093/ageing/afac200, PMID: 36153750 PMC9509667

[ref10] Hahn M. Gröschel S. Gröschel K. Uphaus T. (2023). Association of Delirium Incidence with visitation restrictions due to COVID-19 pandemic in patients with acute cerebrovascular disease in a stroke-unit setting: a retrospective cohort study. Gerontology 69, 273–281. doi: 10.1159/000526165, PMID: 36202083

[ref11] Inouye S. K. Westendorp R. G. J. Saczynski J. S. (2014). Delirium in elderly people. Lancet 383, 911–922. doi: 10.1016/S0140-6736(13)60688-1, PMID: 23992774 PMC4120864

[ref12] Koedam E. L. G. E. Lehmann M. van der Flier W. M. Scheltens P. Pijnenburg Y. A. L. Fox N.. (2011). Visual assessment of posterior atrophy development of a MRI rating scale. Eur. Radiol. 21, 2618–2625. doi: 10.1007/s00330-011-2205-4, PMID: 21805370 PMC3217148

[ref13] Lütz A. Radtke F. M. Franck M. Seeling M. Gaudreau J.-D. Kleinwächter R.. (2008). Die Nursing Delirium Screening Scale (Nu-DESC)—Richtlinienkonforme Ubersetzung für den deutschsprachigen Raum. Anasthesiol. Intensivmed. Notfallmed. Schmerzther. 43, 98–102. doi: 10.1055/s-2008-1060551, PMID: 18293243

[ref14] Nasreddine Z. S. Phillips N. A. Bédirian V. Charbonneau S. Whitehead V. Collin I.. (2005). The Montreal cognitive assessment, MoCA: a brief screening tool for mild cognitive impairment. J. Am. Geriatr. Soc. 53, 695–699. doi: 10.1111/j.1532-5415.2005.53221.x, PMID: 15817019

[ref15] Oldenbeuving A. W. Kort P. L. M. Jansen B. P. W. Algra A. Kappelle L. J. Roks G. (2011). Delirium in the acute phase after stroke: incidence, risk factors, and outcome. Neurology 76, 993–999. doi: 10.1212/WNL.0b013e318210411f, PMID: 21307355

[ref16] Oldenbeuving A. W. Kort P. L. M. van Eck Sluijs J. F. Kappelle L. J. Roks G. (2014). An early prediction of delirium in the acute phase after stroke. J. Neurol. Neurosurg. Psychiatry 85, 431–434. doi: 10.1136/jnnp-2013-304920, PMID: 23744891

[ref17] Pasińska P. Wilk A. Kowalska K. Szyper-Maciejowska A. Klimkowicz-Mrowiec A. (2019). The long-term prognosis of patients with delirium in the acute phase of stroke: PRospective observational POLIsh study (PROPOLIS). J. Neurol. 266, 2710–2717. doi: 10.1007/s00415-019-09471-1, PMID: 31325015 PMC6803586

[ref18] Pasquier F. Leys D. Weerts J. G. Mounier-Vehier F. Barkhof F. Scheltens P. (1996). Inter-and intraobserver reproducibility of cerebral atrophy assessment on MRI scans with hemispheric infarcts. Eur. Neurol. 36, 268–272. doi: 10.1159/000117270, PMID: 8864706

[ref19] Qu J. Chen Y. Luo G. Zhong H. Xiao W. Yin H. (2018). Delirium in the acute phase of ischemic stroke: incidence, risk factors, and effects on functional outcome. J. Stroke Cerebrovasc. Dis. 27, 2641–2647. doi: 10.1016/j.jstrokecerebrovasdis.2018.05.034, PMID: 30172676

[ref20] Rhee J. Y. Colman M. A. Mendu M. Shah S. J. Fox M. D. Rost N. S.. (2022). Associations between stroke localization and delirium: a systematic review and Meta-analysis. J. Stroke Cerebrovasc. Dis. 31:106270. doi: 10.1016/j.jstrokecerebrovasdis.2021.106270, PMID: 34954599 PMC8837688

[ref21] Rhodius-Meester H. F. M. Benedictus M. R. Wattjes M. P. Barkhof F. Scheltens P. Muller M.. (2017). MRI visual ratings of brain atrophy and white matter Hyperintensities across the Spectrum of cognitive decline are differently affected by age and diagnosis. Front. Aging Neurosci. 9:117. doi: 10.3389/fnagi.2017.00117, PMID: 28536518 PMC5422528

[ref22] Ringleb P. Köhrmann M. Jansen O. Berlis A. Fischer U. Laufs U.. Akuttherapie des ischämischen Schlaganfalls, S2e-Leitlinie. Leitlinien für Diagnostik und Therapie in der Neurologie. (2022) [cited 2023 Sep 26]. Available at: www.dgn.org/leitlinien

[ref23] Rollo E. Brunetti V. Scala I. Callea A. Marotta J. Vollono C.. (2022). Impact of delirium on the outcome of stroke: a prospective, observational, cohort study. J. Neurol. 269, 6467–6475. doi: 10.1007/s00415-022-11309-2, PMID: 35945396 PMC9618551

[ref24] Sarraj A. Albers G. W. Mitchell P. J. Hassan A. E. Abraham M. G. Blackburn S.. (2023). Thrombectomy outcomes with general vs nongeneral anesthesia: a pooled patient-level analysis from the EXTEND-IA trials and SELECT study. Neurology 100, e336–e347. doi: 10.1212/WNL.0000000000201384, PMID: 36289001 PMC9869759

[ref25] Scheltens P. Leys D. Barkhof F. Huglo D. Weinstein H. C. Vermersch P.. (1992). Atrophy of medial temporal lobes on MRI in "probable" Alzheimer's disease and normal ageing: diagnostic value and neuropsychological correlates. J. Neurol. Neurosurg. Psychiatry 55, 967–972. doi: 10.1136/jnnp.55.10.967, PMID: 1431963 PMC1015202

[ref26] Shaw R. C. Walker G. Elliott E. Quinn T. J. (2019). Occurrence rate of delirium in acute stroke settings: systematic review and Meta-analysis. Stroke 50, 3028–3036. doi: 10.1161/STROKEAHA.119.025015, PMID: 31554501

[ref27] Shi Q. Presutti R. Selchen D. Saposnik G. (2012). Delirium in acute stroke: a systematic review and meta-analysis. Stroke 43, 645–649. doi: 10.1161/STROKEAHA.111.643726, PMID: 22267831

[ref28] Thomas C. Kreisel S. H. Oster P. Driessen M. Arolt V. Inouye S. K. (2012). Diagnosing delirium in older hospitalized adults with dementia: adapting the confusion assessment method to international classification of diseases, tenth revision, diagnostic criteria. J. Am. Geriatr. Soc. 60, 1471–1477. doi: 10.1111/j.1532-5415.2012.04066.x, PMID: 22881707 PMC3422775

[ref29] Vater V. Olm H.-P. Nydahl P. (2024). Delir bei Schlaganfall: systematisches Review und Metaanalyse. Med Klin Intensivmed Notfmed 119, 49–55. doi: 10.1007/s00063-023-01013-y, PMID: 37166458

[ref30] Zaal I. J. Devlin J. W. Peelen L. M. Slooter A. J. C. (2015). A systematic review of risk factors for delirium in the ICU. Crit. Care Med. 43, 40–47. doi: 10.1097/CCM.0000000000000625, PMID: 25251759

